# Prognosis Between ST-Elevation and Non-ST-elevation Myocardial Infarction in Older Adult Patients

**DOI:** 10.3389/fcvm.2021.749072

**Published:** 2022-01-03

**Authors:** Shih-Sheng Chang, Chiung-Ray Lu, Ke-Wei Chen, Zhe-Wei Kuo, Shao-Hua Yu, Shih-Yi Lin, Hong-Mo Shi, Hei-Tung Yip, Chia-Hung Kao

**Affiliations:** ^1^Division of Cardiovascular Medicine, China Medical University Hospital, Taichung, Taiwan; ^2^School of Medicine, College of Medicine, China Medical University, Taichung, Taiwan; ^3^Graduate Institute of Biomedical Sciences and School of Medicine, College of Medicine, China Medical University, Taichung, Taiwan; ^4^Department of Emergency Medicine, China Medical University Hospital, Taichung, Taiwan; ^5^Division of Nephrology and Kidney Institute, China Medical University Hospital, Taichung, Taiwan; ^6^Management Office for Health Data, China Medical University Hospital, Taichung, Taiwan; ^7^College of Medicine, China Medical University, Taichung, Taiwan; ^8^Department of Nuclear Medicine and PET Center, China Medical University Hospital, Taichung, Taiwan; ^9^Department of Bioinformatics and Medical Engineering, Asia University, Taichung, Taiwan; ^10^Center of Augmented Intelligence in Healthcare, China Medical University Hospital, Taichung, Taiwan

**Keywords:** acute myocardial infarction, STEMI, NSTEMI, older adult, revascularization, outcome

## Abstract

**Background:** Whether there is a difference in prognosis between elderly patients with ST-elevation myocardial infarction (STEMI) and non-ST-elevation myocardial infarction (NSTEMI) remains mysterious.

**Methods:** We conducted a retrospective cohort study by analyzing the data in the Longitudinal Health Insurance Database (LHID) in Taiwan to explore differences between STEMI and NSTEMI with respect to in-hospital and long-term (3-year) outcomes among older adult patients (aged ≥65 years). Patients were further stratified based on whether they received coronary revascularization.

**Results:** In total, 5,902 patients aged ≥65 years with acute myocardial infarction (AMI) who underwent revascularization (2,254) or medical therapy alone (3,648) were included. In the revascularized group, no difference was observed in cardiovascular (CV) and all-cause mortality during hospitalization or at 3-year follow-up between the two AMIs. Conversely, in the non-revascularized group, patients with NSTEMI had higher crude odds ratio (cOR) for all-cause death during hospitalization [cOR: 1.33, 95% confidence interval (CI) = 1.07–1.65] and at 3-year follow-up (cOR: 1.47, 95% CI = 1.21–1.91) relative to patients with STEMI. However, after multivariable adjustments, only NSTEMI indicated fewer in-hospital CV death [adjusted odds ratio (aOR): 0.75, 95% CI = 0.58–0.98] than STEMI in non-revascularized group. Moreover, major bleeding was not different between patients with STEMI or NSTEMI aged ≥65 years old.

**Conclusion:** Classification of AMI is not associated with the difference of in-hospital or 3-year CV and all-cause death in older adult patients received revascularization. In a 3-year follow-up period, STEMI was an independent predictor of a higher incidence of revascularization after the index event. Non-ST-elevation myocardial infarction had more incidence of MACE than patients with STEMI did in both treatment groups.

## Key Points

**What is already known about this subject?**

The patient characteristics and management procedures are quite different between STEMI and NSTEMI. Results of the studies with respect to the short-term and long-term outcomes of these two types of AMIs were inconsistent, and especially data were scarce in the elderly population.

**What might this study add?**

In this retrospective study comprising 5,902 AMI patients aged 65 years and older, no difference was observed in CV and all-cause mortality during hospitalization or at 3-year follow-up between the two AMIs in the revascularized group, but NSTEMI could indicate fewer in-hospital CV death than STEMI in non-revascularized group. Major bleeding was not different between STEMI and NSTEMI patients older than 65 years old.

**How might this impact on clinical practice?**

Our study delineated the prognostic information of STEMI and NSTEMI among old adult patients, which could provide useful information to develop treatment and follow-up strategies.

## Introduction

Acute myocardial infarction (AMI) is a leading cause of hospital admissions and mortality in Taiwan and around the world (1). Rapid diagnosis, treatment, and early revascularization can substantially improve the outcomes of patients with AMI (2–4). Differences in short- and long-term mortalities have been reported between ST-elevation myocardial infarction (STEMI) and non-ST-elevation myocardial infarction (NSTEMI) (5–8). Compared with patients with NSTEMI, those with STEMI encounter greater myocardial damage, which entails an increased risk of early mortality. However, multimorbidity and multivessel coronary disease have been more common in patients with NSTEMI than in patients with STEMI, leading to a higher risk of long-term mortality in patients with NSTEMI (9, 10). Although most studies have reported higher in-hospital fatality rates among patients with STEMI (11) than in patients with NSTEMI, results from the Global Registry of Acute Coronary Events indicated lower post-discharge mortality rates in patients with STEMI than in patients with NSTEMI (12). However, due to the prevalent use of primary percutaneous coronary intervention (PCI) for STEMI, the in-hospital death rate of patients with STEMI has decreased significantly and is now even lower than that of patients with NSTEMI (11). The results of studies regarding the long-term prognosis of these two MI types have remained inconclusive (5, 11–14). Older adults with MI have more risk factors or comorbidities and are considered susceptible to complications from PCI or coronary artery bypass surgery (CABG). Only a few studies have evaluated the differences in the prognosis of STEMI and NSTEMI among older adult patients.

The goal of this study was to investigate the differences in short- and long-term outcomes between STEMI and NSTEMI among older adult patients ≥65 years. This study also determined whether AMI types were associated with major bleeding during hospitalization.

## Methods

### Patient and Public Involvement Statement

The data for this study were obtained from the National Health Insurance Research Database, which contains medical records of >99% of Taiwan's residents that have been documented since 1995. We used a subset of this data set, called the Longitudinal Health Insurance Database (LHID), that contains 1 million randomly selected patients, who were followed up until 2013. The LHID comprises data on outpatient visits, admission records, prescription, and treatment information. Data on diagnosis and therapy were recorded according to the International Classification of Diseases, Ninth Revision and Tenth Revision, Clinical Modification (ICD-9-CM and ICD-10-CM, respectively). This study was approved by the institutional review board of China Medical University Hospital Research Ethics Committee (CMUH104-REC2-115[AR-4]).

### Study Design and Population

This was a retrospective study spanning 13 years. For the study, we included people ≥65 years old who were diagnosed with AMI (ICD-9-CM code 410) at any time between 2000 and 2010. Coronary revascularization was defined as the presence of either PCI or CABG or both. We first divided patients into two groups, namely those who received coronary revascularization, including PCI (ICD-9-CM procedure code 36.0) or CABG (ICD-9-CM procedure code 36.1) and those who did not. Patients in each group were further divided into STEMI (ICD-9-CM codes 410.0–410.5) and NSTEMI (ICD-9-CM codes 410.6–410.9) groups.

### Outcome Measurements and Confounders

The outcome measurements were in-hospital outcomes, in-hospital major bleeding events (revascularized group), and long-term (3 years) outcomes. In-hospital outcomes included mechanical circulatory support (MCS) comprised of extracorporeal membrane oxygenation (ECMO; ICD-9-CM procedure codes 37.62 and 39.65), and intra-aortic balloon pump (IABP; ICD-9-CM procedure code 37.61); congestive heart failure (CHF; ICD-9-CM code 428), stroke (ICD-9-CM codes 430–438), cardiovascular (CV) death, all-cause death, and length of hospital stay, and intensive care unit (ICU) stay. Cardiovascular death was defined as a diagnosis of CV disease within 3 months before the date of death (ICD-9-CM codes 390–459). Three-point major adverse cardiovascular events (three-point MACE) consisted of CHF, stroke, and CV death. The long-term events comprised revascularization after index event, CHF, stroke, recurrent MI, CV death, and death within 3 years after discharge. Four-point MACE were made of CHF, stroke, recurrent MI, and CV death.

We also included the following comorbidities into the analysis: diabetes (ICD-9-CM code 250), hypertension (ICD-9-CM codes 401–405), hyperlipidemia (ICD-9-CM code 272), coronary heart disease (ICD-9-CM code 410–414), cerebrovascular accident (ICD-9-CM codes 430–438), prior MI (ICD-9-CM code 410), prior PCI (ICD-9-CM procedure code 36.0), chronic kidney disease (ICD-9-CM codes 580–589), peripheral arterial occlusion disease (ICD-9-CM codes 440–444), and atrial fibrillation (ICD-9-CM code 427.31). Furthermore, the involvement of related medications, which were aspirin, clopidogrel, angiotensin-converting-enzyme inhibitors/angiotensin II receptor blockers (ACEI/ARB), beta blockers, and statins, was discussed.

### Statistical Analysis

The differences in the distribution of sex, age, comorbidities, and medication between STEMI and NSTEMI groups were examined using a chi-square test and Student's *t*-test. A logistic regression model was applied to estimate the adjusted odds ratio (aOR) of in-hospital outcomes between two types of AMI by including sex, age, and comorbidities. We used a Poisson regression model to compare the difference in the length of hospital stay between the two types of MI. The risk of long-term outcome among STEMI and NSTEMI patients was determined using the Cox regression model.

## Results

We recruited 5,902 patients with AMI aged ≥65 years in this study. Among these patients, 2,254 had received coronary revascularization (STEMI: 966 patients; NSTEMI: 1,288 patients) and 3,648 had not (STEMI: 930 patients; NSTEMI: 2,718 patients). The demographic characteristics of the study population are presented in Table 1. Both treatment groups had a higher proportion of men. Furthermore, the proportions of both sexes were equal in the STEMI and NSTEMI groups among those who were revascularized, but the proportion of women in the NSTEMI group was higher than that in the STEMI group among those who were non-revascularized. Patients with NSTEMI were older than patients with STEMI in both revascularized (NSTEMI: 76.0 ± 6.58 vs. STEMI: 74.6 ± 6.45) and non-revascularized (NSTEMI: 78.4 ± 7.38 vs. STEMI: 76.9 ± 6.87) groups. Notably, in the non-revascularized group, 18.8% of patients were older than 85 years old (13.4% in the STEMI group, and 20.6% in the NSTEMI group), whereas only 9% patients of the revascularized group were ≥85 years old.

**Table 1:** Demographic characteristics of STEMI and NSTEMI in patients over 65-years-old with different treatment strategies.

	**Revascularized**					**Non-revascularized**				
	**STEMI**		**NSTEMI**			**STEMI**		**NSTEMI**		
	***N*** **=** **966**		***N*** **=** **1,288**			***N*** **=** **930**		***N*** **=** **2,718**		
**Variables**	***n***	**%**	***n***	**%**	***p*-values**	***n***	**%**	***n***	**%**	***p*-values**
**Sex**					0.400					0.001
Female	310	32.1	435	33.8		359	38.6	1,216	44.7	
Age^*^					<0.001					<0.001
65–74	545	56.4	614	47.7		379	40.8	935	34.4	
75–84	348	36.0	544	42.2		426	45.8	1,223	45.0	
≥85	73	7.6	130	10.1		125	13.4	560	20.6	
Mean (SD)	74.6	(6.45)	76.0	(6.58)	<0.001	76.9	(4.73)	78.4	(5.14)	<0.001
**Comorbidities**										
Diabetes	429	44.4	726	56.4	<0.001	379	40.8	1,352	49.7	<0.001
Hypertension	775	80.2	1,160	90.1	<0.001	746	80.2	2,352	86.5	<0.001
Hyperlipidemia	460	47.6	767	59.5	<0.001	323	34.7	1,223	45.0	<0.001
CAD	584	60.5	984	76.4	<0.001	567	61.0	1,976	72.7	<0.001
Prior AMI	104	10.8	189	14.7	0.492	109	11.7	341	12.5	0.279
Prior PCI	42	4.3	136	10.6	<0.001	16	1.7	149	5.5	<0.001
Stroke	308	31.9	536	41.6	<0.001	323	34.7	1,258	46.3	<0.001
CKD	135	14.0	342	26.6	<0.001	179	19.2	774	28.5	<0.001
PAOD	97	10.0	261	20.3	<0.001	96	10.3	443	16.3	<0.001
AF	49	5.1	101	7.8	0.010	63	6.8	354	13.0	<0.001
**Medication**										
Aspirin	709	73.4	1,116	86.6	<0.001	625	67.2	2,246	82.6	<0.001
Clopidogrel	128	13.3	373	29.0	<0.001	80	8.6	517	19.0	<0.001
ACEI/ARB	588	60.9	1,017	79.0	<0.001	548	58.9	1,983	73.0	<0.001
Beta blockers	628	65.0	1,021	79.3	<0.001	562	60.4	1,997	73.5	<0.001
Statins	261	27.0	599	46.5	<0.001	162	17.4	867	31.9	<0.001

*CAD, coronary artery disease; AMI, acute myocardial infarction; PCI, percutaneous coronary intervention; CKD, chronic kidney disease; PAOD, peripheral arterial occlusive disease; AF, atrial fibrillation; ACEI, angiotensin-converting-enzyme inhibitors; ARB, angiotensin II receptor blockers*.

* *Examined by Student t-test*.

In both treatment groups, patients with NSTEMI had more comorbidities than patients with STEMI did. In both treatment groups, compared with STEMI patients, NSTEMI patients used more medications before AMI, which included aspirin, clopidogrel, ACEI/ARB, beta blockers, and statins.

Table 2 presents the in-hospital outcomes of the present study. Among patients with revascularization, the aOR of receiving MCS, including ECMO and IABP, in the NSTEMI group was 2.84 relative to the STEMI group [95% confidence interval (CI) = 1.13–7.11]. In the revascularized group, CHF, stroke, CV death, and all-cause death during hospitalization did not differ between the STEMI and NSTEMI groups. In the non-revascularized group, more all-cause deaths were observed among patients with NSTEMI than patients with STEMI. After confounders were controlled for, no more difference observed in all-cause death and other CV endpoints between patients with STEMI and NSTEMI, except for CV death (aOR: 0.75, 95% CI = 0.58–0.98, NSTEMI vs. STEMI). For both treatment strategies, patients in the NSTEMI group had a longer hospital and ICU stay than those in the STEMI group. Among AMI patients aged 65–74 years old, most in-hospital CV outcomes were not different between STEMI and NSTEMI, except for NSTEMI was risk for more MCS in the revascularized group, longer ICU and hospital stay in both treatment groups, and all-cause death (aOR: 1.55, 95% CI = 1.00–2.41) in non-revascularized group when compared to STEMI counterparts. Among AMI patients older than 75 years old, comparing to STEMI patients, NSTEMI ones had longer length of stay of ICU and hospital in both treatment groups and was observed to have fewer CV death (aOR: 0.70, 95% CI = 0.51–0.96) in the non-revascularized group. Nonetheless, other in-hospital CV outcomes were not different between two classes of AMI in both treatment groups (Supplementary Table 1).

**Table 2:** In-hospital outcomes of STEMI and NSTEMI in patients older than 65-year-old with different treatment strategies.

	**Revascularized**							
	**STEMI**		**NSTEMI**		**Univariable analysis**		**Multivariable analysis**	
***All***	***N*** **=** **966**		***N*** **=** **1,288**					
	***n***	**%**	***n***	**%**	**OR (95% CI)**	***p***	**aOR (95% CI)**	***p***
MCS	6	0.62	25	1.94	3.17 (1.29, 7.75)	0.012	2.84 (1.13, 7.11)	0.026
**Outcomes**								
CHF	23	2.38	32	2.48	1.05 (0.61, 1.80)	0.875	0.90 (0.52, 1.58)	0.720
Stroke	12	1.24	16	1.24	1.00 (0.47, 2.12)	>0.99	0.89 (0.41, 1.94)	0.772
CV death	47	4.87	67	5.2	1.07 (0.73, 1.57)	0.718	0.89 (0.60, 1.32)	0.555
Three-point MACE	78	8.07	108	8.39	1.04 (0.77, 1.41)	0.791	0.88 (0.64, 1.21)	0.443
Death	52	5.38	79	6.13	1.15 (0.80, 1.65)	0.451	0.97 (0.66, 1.41)	0.854
	Median	(Q1–Q3)	Median	(Q1–Q3)	RR (95% CI)	*p*	RR (95% CI)	*p*
**Length of stay** ^‡^								
Total	8	(5–13)	10	(6–19)	1.47 (1.44, 1.50)	<0.001	1.37 (1.34, 1.40)	<0.001
ICU	3	(2–6)	4	(3–8)	1.24 (1.21, 1.29)	<0.001	1.17 (1.13, 1.21)	<0.001
	**Non-revascularized**							
	**STEMI**		**NSTEMI**		**Univariable analysis**		**Multivariable analysis**	
***All***	***N*** **=** **930**		***N*** **=** **2,718**					
	***n***	**%**	***n***	**%**	**OR (95% CI)**	***p***	**aOR (95% CI)**	***p***
MCS	0	–	5	0.18				
**Outcomes**								
CHF	28	3.01	83	3.05	1.01 (0.66, 1.57)	0.948	0.93 (0.60, 1.46)	0.763
Stroke	14	1.51	50	1.84	1.23 (0.68, 2.23)	0.504	1.19 (0.65, 2.19)	0.571
CV death	95	10.2	235	8.65	0.83 (0.65, 1.07)	0.150	0.75 (0.58, 0.98)	0.032
Three-point MACE	125	13.4	352	13	0.96 (0.77, 1.19)	0.702	0.88 (0.70, 1.10)	0.268
Death	121	13	450	16.6	1.33 (1.07, 1.65)	0.010	1.19 (0.95, 1.48)	0.132
	Median	(Q1–Q3)	Median	(Q1–Q3)	RR (95% CI)	*p*	RR (95% CI)	*p*
**Length of stay** ^‡^								
Total	9	(5–15)	9	(5–18)	1.20 (1.18, 1.22)	<0.001	1.16 (1.14, 1.18)	<0.001
ICU	4	(3–8)	4	(2–9)	1.18 (1.14, 1.21)	<0.001	1.15 (1.12, 1.19)	<0.001

*MCS, mechanical circulatory support, including ECMO, extracorporeal membrane oxygenation; or IABP, intra-aortic balloon pump; CV, cardiovascular; ICU, intensive care unit; Three-point major adverse cardiovascular events (MACE): heart failure, stroke, and CV death; p, p-value; cOR, crude odds ratio estimated by univariable model; aOR, adjusted odds ratio estimated by multivariable model with controlling for sex, age, and comorbidities*.

‡ *Analyzed by poisson regression model*.

The results in Table 3 demonstrate the bleeding events during hospitalization in STEMI and NSTEMI patients aged ≥65 years. The event of major bleeding between two classes of AMI patients were not significantly different no matter receiving coronary revascularization or not. Furthermore, non-revascularized AMI patients had numerically more in-hospital major bleeding events compared to revascularized counterparts (data not shown).

**Table 3:** Risk of bleeding from revascularization in patients older than 65 years with acute myocardial infarction.

	**STEMI**		**NSTEMI**					
**Age ≥65**	***n***	**%**	***n***	**%**	**cOR (95% CI)**	***p*-values**	**aOR (95% CI)**	***p*-values**
**Revascularized**	*N* = 966		*N* = 1,288					
Major bleeding	5	0.52	8	0.62	1.2 (0.39, 3.68)	0.748	1.20 (0.37, 3.90)	0.764
Gastrointestinal bleeding	5	0.52	4	0.31	0.6 (0.16, 2.24)	0.448	0.69 (0.17, 2.77)	0.603
ICH	0	–	3	0.23				
Other critical site bleeding	0	–	1	0.08				
**Non-revascularized**	*N* = 930		*N* = 2,718					
Major bleeding	9	0.97	33	1.21	1.26 (0.60, 2.64)	0.544	1.18 (0.55, 2.53)	0.679
Gastrointestinal bleeding	6	0.65	29	1.07	1.66 (0.69, 4.01)	0.261	1.54 (0.62, 3.83)	0.351
ICH	2	0.22	2	0.07	0.34 (0.05, 2.44)	0.285	0.39 (0.05, 2.90)	0.357
Other critical site bleeding	1	0.11	2	0.07	0.69 (0.06, 7.57)	0.758	0.82 (0.06, 11.3)	0.882

*ICH, intracerebral hemorrhage; cOR, crude odds ratio estimated by univariable model; aOR, adjusted odds ratio estimated by multivariable model with controlling for sex, age, and comorbidities; Gastrointestinal bleeding (ICD-9-CM codes 530.7, 531, 531.2, 531.4, 531.6, 532, 532.2, 532.4, 532.6, 533, 533.2, 533.4, 533.6, 534, 534.2, 534.4, 534.6, 569.3, 535.01, 535.11, 535.21, 535.31, 535.41, 535.51, 535.61, 535.71, 537.83, 537.84, 562.02, 562.03, 562.12, 562.13, 569.85, and 578); Intracerebral hemorrhage (ICD-9-CM codes 430, 431, 432.0, 432.1, 432.9, 852.0, 852.2, 852.4, and 853.0); and bleeding at other critical sites (ICD-9-CM codes 336.1, 363.6, 372.72, 376.32, 377.42, 379.23, 593.81, 866.01, 866.02, 866.11, 866.12, 719.1, 729.92, 423.0, and 772.5)*.

During long-term (3 years) follow up in both treatment groups, NSTEMI was an independent predictor of fewer rates of revascularization after index event [revascularized group: adjusted hazard ratio (aHR): 0.8, 95% CI = 0.67–0.96; non-revascularized group: aHR: 0.76, 95% CI = 0.60–0.98] (Table 4). In the revascularized group, NSTEMI patients had more incidence of CHF, stroke, recurrent MI, and four-point MACE in comparison with STEMI patients during 3-year follow-up. Similarly, in the non-revascularized group, NSTEMI patients had more long-term incidence of CHF, stroke, four-point MACE, and all-cause death. After multi-variable model analysis with controlling sex, age, and co-morbidities, other than revascularization after index event, STEMI or NSTEMI *per se* did not influence most long-term CV outcomes, except for that NSTEMI indicated more stroke (aHR: 1.27, 95% CI = 1.07–1.52) in revascularized group and more four-point MACE (aHR: 1.13, 95% CI = 1.01–1.27) in non-revascularized group.

**Table 4:** Long-term outcomes (3-year) of STEMI and NSTEMI in patients older than 65-year-old with different treatment strategies.

	**Revascularized**									
	**STEMI**			**NSTEMI**						
	***n***	**PY**	**IR**	***n***	**PY**	**IR**	**cHR (95% CI)**	***p*-values**	**aHR (95% CI)**	***p*-values**
Revascularization after index event	279	17,610	1.58	309	21,106	1.46	0.82 (0.67, 0.97)	0.02	0.80 (0.67, 0.96)	0.02
CHF	310	17,657	1.76	484	19,195	2.52	1.28 (1.11, 1.47)	<0.001	1.10 (0.95, 1.28)	0.20
Stroke	200	20,292	0.99	361	21,634	1.67	1.57 (1.32, 1.86)	<0.001	1.27 (1.07, 1.52)	0.01
Recurrent MI	88	22,374	0.39	150	25,653	0.58	1.38 (1.06, 1.79)	0.02	1.22 (0.93, 1.60)	0.16
CV death	25	32,379	0.08	36	42,995	0.08	1.08 (0.65, 1.80)	0.76	0.73 (0.43, 1.23)	0.24
Four -point MACE	454	16,332	2.78	702	14,511	4.84	1.31 (1.17, 1.48)	<0.001	1.11 (0.99, 1.26)	0.08
Death	49	31,850	0.15	83	41,654	0.20	1.29 (0.91, 1.84)	0.15	1.00 (0.70, 1.44)	>0.99
	**STEMI**			**NSTEMI**						
	***n***	**PY**	**IR**	***n***	**PY**	**IR**	**cHR (95% CI)**	***p*** **-values**	**aHR (95% CI)**	***p*** **-values**
Revascularization after index event	101	14,060	0.72	207	33,362	0.62	0.76 (0.59, 0.96)	0.02	0.76 (0.60, 0.98)	0.03
CHF	255	12,124	2.10	841	24,223	3.47	1.39 (1.21, 1.60)	<0.001	1.16 (1.00, 1.35)	0.05
Stroke	201	12,553	1.60	640	26,209	2.44	1.34 (1.14, 1.57)	<0.001	1.13 (0.96, 1.33)	0.15
Recurrent MI	98	14,633	0.67	310	33,544	0.92	1.24 (0.99, 1.56)	0.06	1.12 (0.88, 1.41)	0.36
CV death	29	29,233	0.10	114	86,179	0.13	1.33 (0.88, 2.00)	0.17	1.21 (0.80, 1.82)	0.38
Four-point MACE	395	8,572	4.61	1,233	14,276	8.64	1.35 (1.20, 1.51)	<0.001	1.13 (1.01, 1.27)	0.04
Death	68	27,508	0.25	274	74,836	0.37	1.47 (1.12, 1.91)	0.005	1.29 (0.98, 1.69)	0.07

*PY, person-years; IR, incidence rate per 100 person-years; p, p-value; CHF, congestive heart failure; CV, cardiovascular; MI, myocardial infarction; Four-point major adverse cardiovascular event (MACE): heart failure, stroke recurrent MI, and CV death; cHR, crude hazard ratio estimated by univariable model; aHR, adjusted hazard ratio estimated by multivariable model with controlling for sex, age, and comorbidities*.

## Discussion

Our study aimed to investigate the difference in CV outcomes between STEMI and NSTEMI in older adult patients (≥65 years). In the revascularized group, we found no difference in CV or all-cause mortality during hospitalization or 3-year follow-up between the two AMIs. In the non-revascularized group, patients with NSTEMI had more all-cause death during hospitalization and 3-year follow-up relative to patients with STEMI. After multivariable adjustment for the non-revascularized group, we found that AMI type was no longer a predictor for in-hospital or long-term all-cause death, though NSTEMI could predict fewer in-hospital CV death. Furthermore, the present study revealed that older adult patients with AMI (≥65 years) had a similar incidence of in-hospital major bleeding between two types of AMI either receiving revascularization or not.

Consistent with previous study, the present study disclosed that patients with NSTEMI were older, were more often men, had more risk factors or comorbidities, including more prior CAD and PCI and took more medication before index events relative to patients with STEMI (9, 13, 14). Our study demonstrated that in the revascularized group, more patients with NSTEMI received MCS compared with patients with STEMI (1.94 vs. 0.62%, aOR: 2.84, *p* = 0.03). Nonetheless, a recent study that analyzed two US national registries of patients who underwent PCI for AMI complicated by cardiogenic shock reported that more MCS was used by patients with STEMI than by patients with NSTEMI (15). Another study analyzing an OPERA registry similarly reported more prevalent IABP placement in patients with STEMI than in patients with NSTEMI (14). The reason for such contradictory results may be that our study comprised older adults; specifically, studies have suggested that the proportion of NSTEMI with the complication of cardiogenic shock increases with age and that the time of urgent revascularization is relatively slower when NSTEMI is combined with shock relative to the STEMI counterpart (16). Our study also showed the ICU and total length of stay were longer among patients with NSTEMI than STEMI patients, regardless of their revascularization status.

However, CHF, stroke, CV death, or three-point MACE during hospitalization did not differ between the two AMIs in both the treatment groups. In the present study, in-hospital CV and overall death did not differ between patients with STEMI and patients with NSTEMI aged ≥65 years receiving coronary revascularization. Similar results were observed in revascularized patients aged 65–74 years and ≥75 years. The OPERA registry that included 2,151 patients with AMI in France reported similar results, that is, no difference in in-hospital mortality between the two AMI categories (14). However, the results of some studies on short-term AMI mortality are different from ours (13, 17); these results have suggested that the short-term mortality of patients with STEMI is higher than that of patients with NSTEMI. Traditionally, STEMI had been considered to be associated with higher mortality rates relative to NSTEMI in the acute phase possibly due to greater mechanical complications, cardiogenic shock, and stent thrombosis in STEMI (14, 18–20). However, with the wide-adoption of primary PCI and MCS, death among patients with STEMI caused by mechanical complication or cardiogenic shock has decreased significantly (18). In addition, there were too few studies have investigated the difference in in-hospital or long-term outcomes between AMI categories among the older adult to draw a solid conclusion. Among the older adult patients, in-hospital mortality of patients with NSTEMI may be higher than that of patients with STEMI due to the presence of risk factors and comorbidities associated with age, which might result in no significant difference in in-hospital death between the two AMI classes. Further studies are required to determine the difference in the outcomes of STEMI and NSTEMI in older adult patients.

Among the older adult patients with AMI, 62% did not receive coronary revascularization and 74.5% of the non-revascularized patients were NSTEMI. Our study found that NSTEMI was an independent predictor of fewer in-hospital CV mortality in this group. In-hospital all-cause death rate was greater in patients with NSTEMI than in patients with STEMI, but no difference was observed after confounders were adjusted for. In addition, NSTEMI consistently indicated fewer in-hospital CV death in non-revascularized patients aged ≥75-year-old but more all-cause death in the non-revascularized patients aged 65–74 years old. Scarce studies have investigated the outcomes of AMI among older adult patients without revascularization. According to a study of the Japan Acute Myocardial Infarction Registry, in-hospital CV and overall mortality of older adult patients with AMI without revascularization were higher in the STEMI group than in the NSTEMI group, which was not in line with our results (21). Older adult patients may not have received coronary revascularization partly due to the high procedural risk related to their frailty or presence of comorbidities. Because non-revascularized NSTEMI patients have more complex comorbidities than their counterparts with STEMI, logically, the cause of death in patients with NSTEMI is likely to be less cardiogenic.

Older adults have an increased risk of both ischemic and bleeding events (22). Furthermore, revascularization itself can increase the risk of in-hospital bleeding (23). In addition to the stress resulting from surgery or PCI, the need for administering heparin during PCI or CABG and dual anti-platelet therapy (DAPT) after stent implantation are the possible reasons underlying bleeding risk. It is highly dangerous for patients if they have just received coronary stenting but cannot use DAPT because of bleeding. Therefore, bleeding risk in older adult patients with AMI is a highly important factor related to the decision of whether to opt for invasive revascularization or conservative medical treatment. We compared bleeding risk between STEMI and NSTEMI patients who were ≥65 years old in both treatment groups. The major bleeding rate was not different between patients with STEMI and NSTEMI in both treatment groups.

At 3-year follow-up, in both treatment groups, STEMI was an independent predictor of a higher incidence of revascularization after the index event. Patients with NSTEMI had more incidence of CHF, stroke, recurrent MI, and four-point MACE than patients with STEMI did in both treatment groups, although recurrent MI in the non-revascularized group did not reach statistical significance (*p* = 0.06). Non-ST-elevation myocardial infarction patients were also found to have more all-cause death in the non-revascularized group. However, after multivariate analysis, no difference was observed in most CV outcomes between the two AMI types, except for that NSTEMI indicated more stroke in revascularized group and four-point MACE in non-revascularized group. Previous studies have reported inconsistent results for long-term prognosis in patients with NSTEMI and STEMI (5, 9, 10, 13). Some studies have reported that STEMI may have a poorer long-term prognosis due to worse short-term outcomes (5, 10, 13). By contrast, in the present study, the difference in CV outcome was not evident between the two AMI categories during hospitalization, which may be one of the reasons for the absence of a significant difference in long-term CV outcomes. According to our findings, STEMI predicts a greater likelihood of coronary revascularization after the index event, which may stem from the principle that cardiologists prefer only to treat infarct-related artery (IRA) in the STEMI circumstances for a long time and further stage PCI for non-IRA lesions possibly leads to greater rate of revascularization after the index AMI than in NSTEMI. Patients with NSTEMI had more CHF, stroke, and four-point MACE at 3-year follow-up in both groups and more deaths in the non-revascularized group, which may be related to the presence of more risk factors and comorbidities. Therefore, according to the multivariate analysis, NSTEMI was not independent risk for long-term CHF, recurrent MI, or death but still risk for stroke in the revascularized group and four-point MACE in the non-revascularized group.

## Limitations

Like other studies on claims databases, this study has certain limitations. The first is an inability to control for all potential confounders due to a lack of, for example, laboratory data, disease severity, total ischemic time, and lifestyle information (e.g., smoking status, obesity); this may result in bias in the prognostic analysis of the two AMI types. Because our study aimed to explore differences between patients with STEMI and NSTEMI, who have relatively similar characteristics, we further divided them into revascularization and non-revascularization groups; this allowed for some confounders to be controlled for.

The study included patients with AMI between 2000 and 2010 in Taiwan, which may be different from current treatment, including the improvement of medical devices and the concept of adoption of PCI and the secondary prevention.

Due to the limitation of database, we could not analyze the use of the first- and second-generation of drug-eluting stents (DES). Since the second generation of drug eluting stents were introduced in Taiwan around 2008–2010 and the first-generation DES were not immediately removed from the market, our study population might be used more first-generation DES than second-generation. Nonetheless, BMS has been used in certain proportion of CAD patients in Taiwan due to the criteria of reimbursement. Therefore, the type of stent used in our study population should be different from the condition in the OPERA and GRACE registry, which could influence the rate of in-stent restenosis and stent thrombosis and consequent the rate of further revascularization, recurrent MI, and even other CV outcomes.

The accuracy of the diagnosis of STEMI and NSTEMI can't be confirmed from our database due to no detail medical records available. However, according to the study of Cheng et al. (24), the positive predictive value of AMI diagnosis of NHIRD was 0.88 using cross-sectional study to find out the corresponding medical records for detailed review. They also reported the diagnostic consistency of comorbidities among AMI patients was 95.9%. Further, the definition of AMI proposed by the Joint European Society of Cardiology/American Heart Association/World Heart Federation Task Force, AMI can be divided into five types (25). The participants of our study could not be differentiated into these five types. ST-elevation myocardial infarction mostly belongs to type 1, whereas NSTEMI may partially belong to type 2; in particular, those who diagnosed as NSTEMI did not receive revascularization (26). Our results revealed that in the non-revascularized group, the overall in-hospital and 3-year mortality rate of NSTEMI was higher than that of STEMI, which was not observed in the revascularized group. However, after confounding factors were controlled for, NSTEMI was not a predictor of short-term and long-term overall mortality but the adjusted OR for in-hospital CV death was lower in patients with NSTEMI in the non-revascularized group.

## Conclusion

Our study demonstrated that no significant difference exists in in-hospital and 3-year all-cause and CV death between the two types of AMIs aged ≥65 years old in both treatment groups, except for NSTEMI predicted fewer in-hospital CV death in the non-revascularized patients. In the future, further research on older adult patients is needed to determine the prognosis of STEMI and NSTEMI with different treatment strategies.

## Data Availability Statement

The datasets presented in this article are not readily available because the dataset used in this study is held by the Taiwan Ministry of Health and Welfare (MOHW). The Ministry of Health and Welfare must approve our application to access this data. Any researcher interested in accessing this dataset can submit an application form to the Ministry of Health and Welfare requesting access. Please contact the staff of MOHW (Email: stcarolwu@mohw.gov.tw) for further assistance. All relevant data are within the paper. Requests to access the datasets should be directed to stcarolwu@mohw.gov.tw.

## Ethics Statement

The studies involving human participants were reviewed and approved by the Institutional Review Board of China Medical University Hospital Research Ethics Committee (CMUH104-REC2-115[AR-4]). Written informed consent for participation was not required for this study in accordance with the national legislation and the institutional requirements.

## Author Contributions

S-SC, C-RL, and C-HK: conception and design. C-HK: provision of study materials. All authors collection and/or assembly of data, data analysis, interpretation, manuscript writing, and final approval of manuscript. All authors contributed substantially to this study and agree with the content of the manuscript.

## Funding

This study was supported in part by Taiwan Ministry of Health and Welfare Clinical Trial Center (MOHW110-TDU-B-212-124004), China Medical University Hospital (DMR-110-089, DMR-110-222, DMR-111-090, and DMR-111-091), MOST Clinical Trial Consortium for Stroke (MOST 110-2321-B-039-003), and Tseng-Lien Lin Foundation, Taichung, Taiwan.

## Conflict of Interest

The authors declare that the research was conducted in the absence of any commercial or financial relationships that could be construed as a potential conflict of interest.

## Publisher's Note

All claims expressed in this article are solely those of the authors and do not necessarily represent those of their affiliated organizations, or those of the publisher, the editors and the reviewers. Any product that may be evaluated in this article, or claim that may be made by its manufacturer, is not guaranteed or endorsed by the publisher.

## References

[1] Moran AE Tzong KY Forouzanfar MH Rothy GA Mensah GA Ezzati M. Variations in ischemic heart disease burden by age, country, and income: the global burden of diseases, injuries, and risk factors 2010 study. Glob Heart. (2014) 9:91–9. 10.1016/j.gheart.2013.12.007 24977114 PMC4071302

[2] Fox CS Evans JC Larson MG Kannel WB Levy D. Temporal trends in coronary heart disease mortality and sudden cardiac death from 1950 to 1999: the Framingham Heart Study. Circulation. (2004) 110:522–7. 10.1161/01.CIR.0000136993.34344.41 15262842

[3] Rosamond WD Chambless LE Heiss G Mosley TH Coresh J Whitsel E. Twenty-two-year trends in incidence of myocardial infarction, coronary heart disease mortality, and case fatality in 4 US communities, 1987–2008. Circulation. (2012) 125:1848–57. 10.1161/CIRCULATIONAHA.111.047480 22420957 PMC3341729

[4] McGovern PG Jacobs DR Jr Shahar E Arnett DK Folsom AR Blackburn H. Trends in acute coronary heart disease mortality, morbidity, and medical care from 1985 through 1997: the Minnesota heart survey. Circulation. (2001) 104:19–24. 10.1161/01.CIR.104.1.19 11435332

[5] Chan MY Sun JL Newby LK Shaw LK Lin M Peterson ED. Long-term mortality of patients undergoing cardiac catheterization for ST-elevation and non-ST-elevation myocardial infarction. Circulation. (2009) 119:3110–7. 10.1161/CIRCULATIONAHA.108.799981 19506116

[6] Chin CT Wang TY Chen AY Mathews R Alexander KP Roe MT. Trends in outcomes among older patients with non-ST-segment elevation myocardial infarction. Am Heart J. (2014) 167:36.e1–42.e1. 10.1016/j.ahj.2013.10.008 24332140

[7] Roe MT Li S Thomas L Wang TY Alexander KP Ohman EM Peterson ED. Long-term outcomes after invasive management for older patients with non-ST-segment elevation myocardial infarction. Circ Cardiovasc Qual Outcomes. (2013) 6:323–32. 10.1161/CIRCOUTCOMES.113.000120 23652734

[8] Roe MT Parsons LS Pollack CV Jr Canto JG Barron HV Every NR. Quality of care by classification of myocardial infarction: treatment patterns for ST-segment elevation vs non-ST-segment elevation myocardial infarction. Arch Intern Med. (2005) 165:1630–6. 10.1001/archinte.165.14.1630 16043682

[9] Allen LA O'Donnell CJ Camargo CA Jr Giugliano RP Lloyd-Jones DM. Comparison of long-term mortality across the spectrum of acute coronary syndromes. Am Heart J. (2006) 151:1065–71. 10.1016/j.ahj.2005.05.019 16644337

[10] Ren L Ye H Wang P Cui Y Cao S Lv S. Comparison of long-term mortality of acute ST-segment elevation myocardial infarction and non-ST-segment elevation acute coronary syndrome patients after percutaneous coronary intervention. Int J Clin Exp Med. (2014) 7:5588–92. 25664077 PMC4307524

[11] Nikus KC Eskola MJ Virtanen VK Harju J Huhtala H Mikkelsson J. Mortality of patients with acute coronary syndromes still remains high: a follow-up study of 1188 consecutive patients admitted to a university hospital. Ann Med. (2007) 39:63–71. 10.1080/08037060600997534 17364452

[12] Goldberg RJ Currie K White K Brieger D Gabriel Steg P Goodman SG. Six-month outcomes in a multinational registry of patients hospitalized with an acute coronary syndrome (the Global Registry of Acute Coronary Events [GRACE]). Am J Cardiol. (2004) 93:288–93. 10.1016/j.amjcard.2003.10.006 14759376

[13] Fokkema ML James SK Albertsson P Aasa M Åkerblom A Calais F. Outcome after percutaneous coronary intervention for different indications: long-term results from the Swedish Coronary Angiography and Angioplasty Registry (SCAAR). EuroIntervention. (2016) 12:303–11. 10.4244/EIJY15M10_07 26485732

[14] Montalescot G Dallongeville J Van Belle E Rouanet S Baulac C Degrandsart A. STEMI and NSTEMI: are they so different? 1 Year outcomes in acute myocardial infarction as defined by the ESC/ACC definition (the OPERA registry). Eur Heart J. (2007) 28:1409–17. 10.1093/eurheartj/ehm031 17412730

[15] Dhruva SS Ross JS Mortazavi BJ Hurley NC Krumholz HM Curtis JP. Use of mechanical circulatory support devices among patients with acute myocardial infarction complicated by cardiogenic shock. JAMA Netw Open. (2021) 4:e2037748. 10.1001/jamanetworkopen.2020.37748 33616664 PMC7900859

[16] Anderson ML Peterson ED Peng SA Wang TY Ohman EM Bhatt DL. Differences in the profile, treatment, and prognosis of patients with cardiogenic shock by myocardial infarction classification: a report from NCDR. Circ Cardiovasc Qual Outcomes. (2013) 6:708–15. 10.1161/CIRCOUTCOMES.113.000262 24221834

[17] Steg PG Goldberg RJ Gore JM Fox KA Eagle KA Flather MD. Baseline characteristics, management practices, and in-hospital outcomes of patients hospitalized with acute coronary syndromes in the Global Registry of Acute Coronary Events (GRACE). Am J Cardiol. (2002) 90:358–63. 10.1016/S0002-9149(02)02489-X 12161222

[18] Aissaoui N Puymirat E Delmas C Ortuno S Durand E Bataille V. Trends in cardiogenic shock complicating acute myocardial infarction. Eur J Heart Fail. (2020) 22:664–72. 10.1002/ejhf.1750 32078218

[19] Liu SP Shibu MA Tsai FJ Hsu YM Tsai CH Chung JG. Tetramethylpyrazine reverses high-glucose induced hypoxic effects by negatively regulating HIF-1α induced BNIP3 expression to ameliorate H9c2 cardiomyoblast apoptosis. Nutr Metab (Lond). (2020) 17:12. 10.1186/s12986-020-0432-x 32021640 PMC6995207

[20] Elbadawi A Elgendy IY Mahmoud K Barakat AF Mentias A Mohamed AH. Temporal trends and outcomes of mechanical complications in patients with acute myocardial infarction. JACC Cardiovasc Interv. (2019) 12:1825–36. 10.1016/j.jcin.2019.04.039 31537282

[21] Suzuki M Nishihira K Takegami M Honda S Kojima S Takayama M. Clinical profiles and outcomes in the treatment of acute myocardial infarction in Japan of aging society. Heart Vessels. (2020) 35:1681–8. 10.1007/s00380-020-01654-5 32601976

[22] Riobóo-Lestón L Raposeiras-Roubin S Abu-Assi E Iñiguez-Romo A. Bleeding risk assessment in elderly patients with acute coronary syndrome. J Geriatr Cardiol. (2019) 16:145–50. 10.11909/j.issn.1671-5411.2019.02.002 30923546 PMC6431601

[23] Simonsson M Wallentin L Alfredsson J Erlinge D Hellström Ängerud K Hofmann R. Temporal trends in bleeding events in acute myocardial infarction: insights from the SWEDEHEART registry. Eur Heart J. (2020) 41:833–43. 10.1093/eurheartj/ehz593 31504404

[24] Cheng CL Lee CH Chen PS Li YH Lin SJ Yang YHK. Validation of acute myocardial infarction cases in the National Health Insurance Research Database in Taiwan. J Epidemiol. (2014) 24:500–7. 10.2188/jea.JE20140076 25174915 PMC4213225

[25] Thygesen K Alpert JS Jaffe AS Chaitman BR Bax JJ Morrow DA. Fourth universal definition of myocardial infarction 2018. Eur Heart J. (2018), 40: 237–69. 10.1161/CIR.0000000000000617 30165617

[26] Sandoval Y Thygesen K. Myocardial infarction type 2 and myocardial injury. Clin Chem. (2017) 63:101–7. 10.1373/clinchem.2016.255521 28062614

