# Supplementary material for: Prognosis Between ST-Elevation and Non-ST-elevation Myocardial Infarction in Older Adult Patients
Source: Front Cardiovasc Med. 2022 Jan 3;8:749072. doi: 10.3389/fcvm.2021.749072 (PMC8761910; doi:10.3389/fcvm.2021.749072)
Supplement: Supplementary file 1 [file Table_1.DOCX]

Supplement Table S1. In-hospital outcomes of STEMI and NSTEMI in patients older than 65-year-old with different treatment strategies.

|  | **Revascularized** | | | | | | | | | | | | | | | |
| --- | --- | --- | --- | --- | --- | --- | --- | --- | --- | --- | --- | --- | --- | --- | --- | --- |
|  | **STEMI** | |  | | **NSTEMI** | | | | **Univariable analysis** | | | | **Multivariable analysis** | | | |
| ***65-74*** | **N=545** | |  | | **N=614** | | | |  | |  | |  | |  | |
|  | n | % |  | | n | | % | | cOR (95% CI) | | *p* | | aOR (95% CI) | | *p* | |
| MCS | 2 | 0.37 |  | | 18 | | 2.93 | | 8.20 (1.89,35.49) | | 0.005 | | 7.18 (1.58,32.5) | | 0.011 | |
| Outcomes |  |  |  | |  | |  | |  | |  | |  | |  | |
| CHF | 10 | 1.83 |  | | 11 | | 1.79 | | 0.98 (0.41,2.32) | | 0.956 | | 0.82 (0.32,2.08) | | 0.673 | |
| stroke | 3 | 0.55 |  | | 11 | | 1.79 | | 3.29 (0.91,11.9) | | 0.068 | | 2.83 (0.75,10.7) | | 0.125 | |
| CV death | 14 | 2.57 |  | | 25 | | 4.07 | | 1.61 (0.83,3.13) | | 0.160 | | 1.42 (0.69,2.94) | | 0.343 | |
| 3-point MACE | 26 | 4.77 |  | | 41 | | 6.68 | | 1.43 (0.86,2.37) | | 0.167 | | 1.30 (0.76,2.24) | | 0.339 | |
| death | 17 | 3.12 |  | | 29 | | 4.72 | | 1.54 (0.84,2.83) | | 0.166 | | 1.32 (0.68,2.57) | | 0.408 | |
|  | median | (Q1-Q3) |  | | median | | (Q1-Q3) | | RR (95% CI) | | *p* | | RR (95% CI) | | *p* | |
| Length of stay‡ |  |  |  | |  | |  | |  | |  | |  | |  | |
| total | 8 | (5-12) |  | | 9 | | (6-18) | | 1.34 (1.31, 1.37) | | <0.001 | | 1.33 (1.29,1.37) | | <0.001 | |
| ICU | 3 | (2-5) |  | | 4 | | (2-7) | | 1.20 (1.17, 1.24) | | <0.001 | | 1.13 (1.10,1.19) | | <0.001 | |
| ***≧75*** | **N=421** | |  | | **N=674** | | | |  | |  | |  | |  | |
|  | n | % |  | | n | | % | | cOR (95% CI) | | *p* | | aOR (95% CI) | | *p* | |
| MCS | 4 | 0.95 |  | | 7 | | 1.04 | | 1.09 (0.32,3.76) | | 0.887 | | 1.03 (0.28,3.82) | | 0.963 | |
| Outcomes |  |  |  | |  | |  | |  | |  | |  | |  | |
| CHF | 13 | 3.09 |  | | 21 | | 3.12 | | 1.01 (0.50,2.04) | | 0.979 | | 0.91 (0.44,1.89) | | 0.800 | |
| stroke | 9 | 2.14 |  | | 5 | | 0.74 | | 0.34 (0.11,1.03) | | 0.056 | | 0.34 (0.11,1.08) | | 0.066 | |
| CV death | 33 | 7.84 |  | | 42 | | 6.23 | | 0.78 (0.49,1.25) | | 0.307 | | 0.68 (0.41,1.11) | | 0.125 | |
| 3-point MACE | 52 | 12.35 |  | | 67 | | 9.94 | | 0.78 (0.53,1.15) | | 0.213 | | 0.71 (0.47,1.06) | | 0.095 | |
| death | 35 | 8.31 |  | | 50 | | 7.42 | | 0.88 (0.56,1.39) | | 0.590 | | 0.79 (0.49,1.26) | | 0.320 | |
|  | median | (Q1-Q3) |  | | median | | (Q1-Q3) | | RR (95% CI) | | *p* | | RR (95% CI) | | *p* | |
| Length of stay‡ |  |  |  | |  | |  | |  | |  | |  | |  | |
| total | 8 | (6-14) |  | | 10 | | (7-20) | | 1.43 (1.39,1.48) | | <0.001 | | 1.34 (1.29, 1.41) | | <0.001 | |
| ICU | 4 | (2-6) |  | | 5 | | (3-9) | | 1.41 (1.37,1.46) | | <0.001 | | 1.32 (1.26, 1.38) | | <0.001 | |
|  | **Non-revascularized** | | | | | | | | | | | | | | |  |
|  | **STEMI** | |  | **NSTEMI** | | | | **Univariable analysis** | | | | **Multivariable analysis** | | | |  |
| ***65-74*** | **N=379** | |  | **N=935** | | | |  | |  | |  | |  | |  |
|  | n | % |  | n | | % | | cOR (95% CI) | | *p* | | aOR (95% CI) | | *p* | |  |
| MCS | 0 | 0.00 |  | 2 | | 0.21 | |  | |  | |  | |  | |  |
| Outcomes |  |  |  |  | |  | |  | |  | |  | |  | |  |
| CHF | 4 | 1.06 |  | 24 | | 2.57 | | 2.47 (0.85,7.17) | | 0.096 | | 2.35 (0.80,6.94) | | 0.122 | |  |
| stroke | 4 | 1.06 |  | 16 | | 1.71 | | 1.63 (0.54,4.91) | | 0.384 | | 1.73 (0.56,5.37) | | 0.341 | |  |
| CV death | 25 | 6.60 |  | 63 | | 6.74 | | 1.02 (0.63,1.65) | | 0.926 | | 1.00 (0.61,1.64) | | >0.99 | |  |
| 3-point MACE | 30 | 7.92 |  | 100 | | 10.70 | | 1.39 (0.91,2.13) | | 0.128 | | 1.40 (0.90,2.18) | | 0.134 | |  |
| death | 29 | 7.65 |  | 113 | | 12.09 | | 1.66 (1.08,2.54) | | 0.020 | | 1.55 (1.00,2.41) | | 0.049 | |  |
|  | median | (Q1-Q3) |  | median | | (Q1-Q3) | | RR (95% CI) | | *p* | | RR (95% CI) | | *p* | |  |
| Length of stay‡ |  |  |  |  | |  | |  | |  | |  | |  | |  |
| total | 8 | (5-12) |  | 9 | | (5-16) | | 1.21 (1.17,1.25) | | <0.001 | | 1.03 (1.21,1.36) | | <0.001 | |  |
| ICU | 4 | (3-5) |  | 4 | | (2-8) | | 1.17 (1.14,1.21) | | <0.001 | | 1.25 (1.19,1.33) | | <0.001 | |  |
| ***≧75*** | **N=551** | |  | **N=1783** | | | |  | |  | |  | |  | |  |
|  | n | % |  | n | | % | | cOR (95% CI) | | *p* | | aOR (95% CI) | | *p* | |  |
| MCS | 0 | 0.00 |  | 3 | | 0.17 | |  | |  | |  | |  | |  |
| Outcomes |  |  |  |  | |  | |  | |  | |  | |  | |  |
| CHF | 24 | 4.36 |  | 59 | | 3.31 | | 0.75 (0.46,1.22) | | 0.247 | | 0.74 (0.45,1.22) | | 0.239 | |  |
| stroke | 10 | 1.81 |  | 34 | | 1.91 | | 1.05 (0.52,2.14) | | 0.890 | | 1.15 (0.55,2.39) | | 0.712 | |  |
| CV death | 70 | 12.70 |  | 172 | | 9.65 | | 0.73 (0.55,0.99) | | 0.040 | | 0.70 (0.51,0.96) | | 0.024 | |  |
| 3-point MACE | 95 | 17.24 |  | 252 | | 14.13 | | 0.79 (0.61,1.02) | | 0.074 | | 0.77 (0.59,1.01) | | 0.060 | |  |
| death | 92 | 16.70 |  | 337 | | 18.90 | | 1.16 (0.90,1.50) | | 0.243 | | 1.09 (0.84,1.42) | | 0.511 | |  |
|  | median | (Q1-Q3) |  | median | | (Q1-Q3) | | RR (95% CI) | | *p* | | RR (95% CI) | | *p* | |  |
| Length of stay‡ |  |  |  |  | |  | |  | |  | |  | |  | |  |
| total | 9 | (5-18) |  | 10 | | (5-19) | | 1.17 (1.15,1.20) | | <0.001 | | 1.14 (1.12, 1.17) | | <0.001 | |  |
| ICU | 4 | (3-10 |  | 5 | | (2-10) | | 1.10 (1.06, 1.14) | | <0.001 | | 1.10 (1.06, 1.15) | | <0.001 | |  |

MCS: mechanical circulatory support, including ECMO (extracorporeal membrane oxygenation) or IABP (intra-aortic balloon pump); CV: cardiovascular; ICU: intensive care unit; 3-point major adverse cardiovascular events (MACE): heart failure, stroke and CV death;
*p*: p-value;
cOR: crude odds ratio estimated by univariable model;
aOR: adjusted odds ratio estimated by multivariable model with controlling for sex, age and comorbidities;
‡: analysed by Poisson regression model
